# Cardiovascular Outcomes of SGLT-2 Inhibitors in Hypertensive Patients With and Without Diabetes: A Retrospective Observational Study

**DOI:** 10.7759/cureus.94467

**Published:** 2025-10-13

**Authors:** Salar Sohail, Talat Bashir, Hajra Tariq, Tayyaba Maheen, Ayesha Asghar, Mukhayya Djumaniyazova, Jawad Hameed, Razwan Ashraf

**Affiliations:** 1 Medicine, HITEC Institute of Medical Sciences, Taxila, PAK; 2 Internal Medicine, Medicare Hospital, Vehari, PAK; 3 Cardiology, Abbottabad International Medical Institute, Abbottabad, PAK; 4 Medicine, NUST School of Health Sciences, Islamabad, PAK; 5 Medicine, Al-Shifa Hospital, Nowshera Virkan, PAK; 6 Pedagogy and Psychology, Urgench State University, Urgench, UZB; 7 Anesthesia and Critical Care, Lady Reading Hospital MTI, Peshawar, PAK; 8 General Practice, Aziz Bhatti Shaheed Teaching Hospital, Gujrat, PAK

**Keywords:** blood pressure control, cardiovascular outcomes, hypertension, sglt-2 inhibitors, type 2 diabetes mellitus

## Abstract

Background

Hypertension, with or without type 2 diabetes mellitus (T2DM), is a major contributor to cardiovascular morbidity and mortality. Sodium-glucose cotransporter 2 (SGLT-2) inhibitors, originally developed for glycemic control, have demonstrated cardioprotective benefits. However, real-world data on their cardiovascular outcomes in hypertensive patients with and without diabetes remain limited.

Objectives

This study aimed to assess whether SGLT-2 inhibitor therapy improves blood pressure control and reduces cardiovascular events in hypertensive patients and to compare these outcomes between those with and without T2DM.

Methodology

This retrospective observational study was conducted at the HITEC Institute of Medical Sciences, Taxila, over 12 months. Medical records of 200 hypertensive patients prescribed SGLT-2 inhibitors were reviewed and categorized into two groups: diabetics (n = 100) and non-diabetics (n = 100). Baseline demographics, comorbidities, laboratory findings, and concomitant medications were recorded. To minimize confounding from other drugs, only patients on stable antihypertensive regimens for at least six months prior to SGLT-2 inhibitor initiation were included. Blood pressure changes were assessed from baseline to six months, and cardiovascular outcomes (hospitalization for heart failure, myocardial infarction, and all-cause mortality) were evaluated using hospital records. Statistical analysis involved paired t-tests, chi-square tests, and Cox regression models.

Results

The mean age of the participants was 56.4 ± 9.8 years, with 58% males. BMI was significantly higher in diabetics than in non-diabetics (29.2 ± 4.1 vs. 27.5 ± 3.9 kg/m², p = 0.01). As expected, diabetics had higher fasting glucose and HbA1c (p < 0.001), while lipid and renal profiles were comparable. After six months, both groups showed significant reductions in systolic and diastolic blood pressure (p < 0.001), with greater systolic reduction in diabetics (-12.5 vs. -9.7 mmHg, p = 0.04). During follow-up, diabetics experienced more cardiovascular events than non-diabetics: hospitalization for heart failure (6% vs. 3%), myocardial infarction (5% vs. 2%), and all-cause mortality (5% vs. 2%). Cox regression identified higher baseline HbA1c and longer hypertension duration as independent predictors of adverse events.

Conclusion

SGLT-2 inhibitors significantly improve blood pressure control and reduce cardiovascular events in hypertensive patients, regardless of diabetic status. However, patients with diabetes exhibit a higher residual risk, highlighting the need for integrated management strategies addressing glycemic and cardiovascular risk factors. The retrospective design allowed real-world evaluation of outcomes, though prospective studies are warranted to confirm these findings.

## Introduction

Cardiovascular disease (CVD) remains the leading cause of morbidity and mortality worldwide and is strongly linked to metabolic disorders such as diabetes mellitus and hypertension [1]. Type 2 diabetes mellitus (T2DM), which accounts for nearly 90% of all diabetes cases, markedly increases the risk of adverse cardiovascular outcomes, including myocardial infarction, stroke, and heart failure [2]. Hypertension, often coexisting with diabetes, independently contributes to this burden by accelerating atherosclerosis and vascular injury [3]. Together, diabetes and hypertension represent a major challenge for cardiovascular health, underscoring the importance of therapeutic strategies that can simultaneously address metabolic and cardiovascular risk.

Sodium-glucose cotransporter 2 (SGLT-2) inhibitors, initially developed as antihyperglycemic agents, act by inhibiting glucose reabsorption in the proximal renal tubules, thereby promoting glucosuria and lowering blood glucose in an insulin-independent manner [4]. Beyond their role in glycemic management, these agents have demonstrated additional benefits, including modest reductions in blood pressure, body weight, and improvements in renal outcomes [5]. More importantly, large cardiovascular outcome trials (CVOTs), such as EMPA-REG OUTCOME, CANVAS, and DECLARE-TIMI 58, have revealed that SGLT-2 inhibitors reduce major adverse cardiovascular events (MACE), hospitalization for heart failure, and cardiovascular mortality [6,7]. These findings have shifted the therapeutic scope of SGLT-2 inhibitors from diabetes control to cardiometabolic protection.

Recent evidence also highlights their benefits in patients without diabetes. The previous trials demonstrated significant reductions in heart failure hospitalizations and cardiovascular deaths in both diabetic and non-diabetic populations treated with SGLT-2 inhibitors [8,9]. Their effects on hemodynamics, arterial stiffness, and renal protection further support a role in managing hypertensive patients, regardless of glycemic status [10]. However, while the cardiovascular benefits of SGLT-2 inhibitors in diabetics are well established, limited data exist regarding their real-world outcomes in hypertensive patients with and without diabetes, particularly in populations outside randomized trial settings.

Given the high prevalence of hypertension, with or without coexisting diabetes, and its central role in cardiovascular morbidity, it is essential to evaluate whether SGLT-2 inhibitors provide comparable cardiovascular benefits across these subgroups. This study was therefore designed to address this gap. Understanding the cardiovascular outcomes of SGLT-2 inhibitors in hypertensive patients, irrespective of diabetic status, may guide more personalized and broader use of these agents in clinical practice. The objective of this study was to explore and compare the cardiovascular outcomes associated with SGLT-2 inhibitor use in hypertensive patients with and without diabetes. 

## Materials and methods

Study design and setting

This retrospective observational study was conducted at the HITEC Institute of Medical Sciences, Taxila, over a period of 12 months from July 2023 to June 2024. The design was chosen to assess clinical outcomes based on existing patient records, thereby providing real-world evidence regarding the cardiovascular benefits of SGLT-2 inhibitors in routine clinical practice.

Study population

The study population included adult hypertensive patients aged between 30 and 75 years, with or without a confirmed diagnosis of T2DM, who had been prescribed an SGLT-2 inhibitor for at least six months. Patient data were retrieved from the institutional database and electronic medical records of the affiliated teaching hospital.

Sample size calculation

The sample size was determined using the World Health Organization (WHO) sample size calculator for observational studies. With a 95% confidence level, a power of 80%, and an expected effect size of 0.35 based on previously published literature examining cardiovascular outcomes of SGLT-2 inhibitors [1], the minimum required sample size was calculated to be 180 participants. To accommodate incomplete or missing data, the final target sample size was increased to 200 patients.

The formula applied was:

$n=(〖(Z(1-&alpha;/2)+Z(1-&beta;))〗^2&times;2&times;\sigma^2)/∆^2$

where n represents the sample size per group, Z1−α/2 is the standard normal deviate at a 95% confidence level (1.96), Z1−β is the standard normal deviate at 80% power (0.84), σ is the assumed standard deviation based on prior studies, and Δ is the minimum clinically significant difference, considered here as 0.35 standard deviations. Substituting these values yielded a required sample size of approximately 180 patients.

Sampling technique

A consecutive sampling technique was employed. All eligible patients who fulfilled the inclusion criteria during the study period were enrolled until the required sample size was achieved.

Inclusion and exclusion criteria

The inclusion criteria comprised adults aged 30 to 75 years diagnosed with hypertension, with or without T2DM, who had been on regular therapy with empagliflozin, dapagliflozin, or canagliflozin for a minimum of six months and had complete clinical and biochemical records available. Patients with type 1 diabetes mellitus, those with secondary causes of hypertension, individuals with advanced chronic kidney disease defined as an estimated glomerular filtration rate below 30 mL/min/1.73 m², those with incomplete medical records, and patients who discontinued SGLT-2 inhibitor therapy within six months of initiation were excluded from the study.

Data collection

Data collection was carried out through a review of medical records. Demographic details such as age, sex, BMI, smoking status, and family history of CVDs were recorded. Clinical history, including duration of hypertension, presence of diabetes, and coexisting comorbidities, was obtained. Treatment details, including the type and dose of SGLT-2 inhibitor and concomitant antihypertensive or antidiabetic medications, were also documented. Cardiovascular outcomes were assessed in terms of blood pressure control, hospitalization due to heart failure, occurrence of myocardial infarction, ischemic stroke, and all-cause mortality. Baseline and follow-up laboratory investigations including fasting blood glucose, HbA1c (in diabetic patients), serum creatinine, lipid profile, and electrolytes were collected to evaluate the metabolic effects of SGLT-2 inhibitors.

Outcome assessment

Outcome assessment involved comparison of baseline cardiovascular and metabolic parameters before the initiation of SGLT-2 inhibitors with those recorded after a minimum of six months of therapy. Blood pressure control was assessed using mean systolic and diastolic blood pressure values averaged from three separate clinic visits. Cardiovascular events such as myocardial infarction, ischemic stroke, and heart failure requiring hospitalization were confirmed through discharge summaries, electrocardiographic records, and imaging reports. All-cause mortality was determined using hospital mortality records or, where necessary, through follow-up telephonic verification.

Statistical analysis

Statistical analysis was performed using the IBM SPSS Statistics for Windows, Version 26.0 (Released 2018; IBM Corp., Armonk, NY, USA). Continuous variables including age, blood pressure, and biochemical parameters were expressed as mean ± standard deviation, while categorical variables such as gender, diabetes status, and cardiovascular events were presented as frequencies and percentages. Comparisons between hypertensive patients with and without diabetes were conducted using the independent sample t-test for continuous variables and the chi-square test for categorical variables. Within-group comparisons of baseline and post-treatment parameters were carried out using paired sample t-tests. Kaplan-Meier survival analysis was applied to assess time-to-event cardiovascular outcomes, and Cox proportional hazards regression was used to identify independent predictors of adverse cardiovascular events. A p-value of less than 0.05 was considered statistically significant.

Ethical considerations

Ethical approval for this retrospective study and patient consent were waived. However, patient confidentiality was strictly maintained by anonymizing all data during collection and analysis.

## Results

A total of 200 hypertensive patients fulfilled the eligibility criteria and were included in the final analysis. Among them, 100 patients (50%) had hypertension \with T2DM (DM group), while the remaining 100 patients had hypertension without diabetes (non-DM group). The mean duration of follow-up was 11.2 ± 1.4 months.

Baseline demographic and clinical characteristics

The baseline demographic and clinical parameters of the study population are summarized in Table 1. The mean age of participants was 56.4 ± 9.8 years, with no significant difference between the DM and non-DM groups (p = 0.27). Male patients represented 58% of the study population. BMI was significantly higher in the DM group (29.2 ± 4.1 kg/m²) compared with the non-DM group (27.5 ± 3.9 kg/m², p = 0.01). The prevalence of dyslipidemia and smoking was also higher among the DM group, although the difference did not reach statistical significance.

**Table 1:** Baseline demographic and clinical characteristics of the study population *Statistically significant at p < 0.05. HTN: hypertension, DM: hypertension with type 2 diabetes mellitus group, non-DM: hypertension without type 2 diabetes mellitus group.

Variable	Total (n = 200)	DM group (n = 100)	Non-DM group (n = 100)	p-value
Age (years, mean ± SD)	56.4 ± 9.8	57.1 ± 9.6	55.6 ± 10.0	0.27
Male sex, n (%)	116 (58)	61 (61)	55 (55)	0.39
BMI (kg/m², mean ± SD)	28.4 ± 4.2	29.2 ± 4.1	27.5 ± 3.9	0.01*
Duration of HTN (years)	8.6 ± 5.1	9.3 ± 5.2	7.9 ± 5.0	0.11
Smoking, n (%)	58 (29)	34 (34)	24 (24)	0.12
Dyslipidemia, n (%)	74 (37)	42 (42)	32 (32)	0.15

Baseline laboratory parameters

Baseline laboratory findings are presented in Table 2. Patients in the DM group had significantly higher fasting blood glucose and HbA1c levels, as expected (p < 0.001). Serum creatinine, lipid profile, and electrolyte levels were comparable between the two groups, with no significant differences.

**Table 2:** Baseline laboratory parameters of the study population *Statistically significant at p < 0.05. LDL: low-density lipoprotein, HDL: high-density lipoprotein, DM: hypertension with type 2 diabetes mellitus group, non-DM: hypertension without type 2 diabetes mellitus group.

Parameter	DM group (n = 100)	Non-DM group (n = 100)	p-value
Fasting blood glucose (mg/dL)	152.3 ± 28.7	99.5 ± 14.8	<0.001*
HbA1c (%)	8.2 ± 1.1	5.7 ± 0.4	<0.001*
Serum creatinine (mg/dL)	1.08 ± 0.24	1.05 ± 0.21	0.47
Total cholesterol (mg/dL)	192.4 ± 34.2	187.9 ± 32.7	0.36
LDL cholesterol (mg/dL)	116.2 ± 24.1	112.7 ± 23.9	0.31
HDL cholesterol (mg/dL)	43.5 ± 7.6	44.1 ± 7.2	0.56
Triglycerides (mg/dL)	154.7 ± 38.3	148.6 ± 37.1	0.27
Serum sodium (mmol/L)	139.2 ± 3.4	139.5 ± 3.1	0.49
Serum potassium (mmol/L)	4.5 ± 0.4	4.6 ± 0.3	0.21

Blood pressure control after six months of SGLT-2 inhibitor therapy

Blood pressure outcomes before and after six months of SGLT-2 inhibitor therapy are presented in Table 3. Both groups demonstrated a statistically significant reduction in systolic and diastolic blood pressure compared with baseline (p < 0.001 within groups). The mean reduction in systolic BP was greater in the DM group (-12.5 mmHg) compared with the non-DM group (-9.7 mmHg, p = 0.04).

**Table 3:** Comparison of blood pressure before and after SGLT-2 inhibitor therapy *Statistically significant at p < 0.05. SGLT-2: sodium-glucose cotransporter 2, BP: blood pressure, DM: hypertension with type 2 diabetes mellitus group, non-DM: hypertension without type 2 diabetes mellitus group.

Parameter	DM group, baseline	DM group, six months	Non-DM, baseline	Non-DM, six months	p-value (between groups)
Systolic BP (mmHg)	146.8 ± 12.3	134.3 ± 10.8	145.5 ± 11.7	135.8 ± 9.9	0.04*
Diastolic BP (mmHg)	89.7 ± 6.8	82.6 ± 6.1	88.9 ± 6.5	82.9 ± 6.3	0.61

Cardiovascular events and mortality

The incidence of cardiovascular events during follow-up is detailed in Table 4. Hospitalization for heart failure was observed in 6% of the DM group and 3% of the non-DM group, while myocardial infarction occurred in 5% and 2% of patients, respectively. Stroke incidence was slightly higher in the DM group, but the difference was not statistically significant. All-cause mortality was 5% in the DM group compared with 2% in the non-DM group. Kaplan-Meier survival analysis demonstrated better event-free survival in the non-DM group (log-rank p = 0.04).

**Table 4:** Cardiovascular events and mortality during follow-up. HF: heart failure, DM: hypertension with type 2 diabetes mellitus group, non-DM: hypertension without type 2 diabetes mellitus group.

Outcome	DM group (n = 100)	Non-DM group (n = 100)	p-value
Hospitalization for HF, n (%)	6 (6)	3 (3)	0.3
Myocardial infarction, n (%)	5 (5)	2 (2)	0.25
Ischemic stroke, n (%)	3 (3)	1 (1)	0.31
All-cause mortality, n (%)	5 (5)	2 (2)	0.25

Predictors of adverse cardiovascular events

Cox proportional hazard regression identified higher baseline HbA1c (HR 1.35, 95% CI: 1.12-1.61, p = 0.002) and longer duration of hypertension (HR 1.08 per year, 95% CI: 1.01-1.16, p = 0.02) as independent predictors of cardiovascular events. Use of SGLT-2 inhibitors was associated with a protective effect on heart failure hospitalization across both groups.

## Discussion

This study explored the cardiovascular outcomes of SGLT-2 inhibitors in hypertensive patients with and without T2DM. A total of 200 patients were followed over 12 months, and findings indicated that SGLT-2 inhibitors improved blood pressure control, reduced cardiovascular events, and lowered heart failure hospitalizations, with variations observed between diabetic and non-diabetic groups.

Baseline demographics and clinical characteristics

In our study population, the mean age was 56.4 years, and the majority were male (58%). Patients in the diabetic group exhibited a significantly higher BMI compared to the non-diabetic group. These findings are consistent with previous reports suggesting that obesity is more prevalent among patients with T2DM and contributes to the clustering of cardiovascular risk factors [6]. The higher prevalence of dyslipidemia and smoking in the diabetic group, although not statistically significant, aligns with epidemiological studies demonstrating that diabetic patients tend to present with multiple cardiovascular risk factors compared with their non-diabetic counterparts [11].

Baseline laboratory parameters

As expected, patients in the diabetic group had significantly elevated fasting blood glucose and HbA1c levels. However, other laboratory markers, including serum creatinine and lipid profiles, were comparable across groups. The similarity in renal function between groups at baseline reflects the careful exclusion of patients with advanced chronic kidney disease, in line with our methodology. These results mirror findings from large-scale outcome trials where baseline renal function was preserved in most participants, allowing clearer assessment of cardiovascular outcomes. Additionally, while SGLT-2 inhibitors have demonstrated favorable effects on triglycerides and HDL in some studies [12], our baseline data did not show major intergroup differences, reinforcing that improvements in metabolic parameters are more likely to be observed after therapy initiation rather than at baseline.

Blood pressure reduction

One of the most consistent findings of this study was the significant reduction in both systolic and diastolic blood pressure after six months of SGLT-2 inhibitor therapy. The decrease was slightly more pronounced in the diabetic group (-12.5 mmHg systolic) compared to the non-diabetic group (-9.7 mmHg systolic). These results are in agreement with previous meta-analyses and randomized controlled trials, which demonstrated modest but clinically meaningful reductions in blood pressure among patients treated with SGLT-2 inhibitors [8]. The hemodynamic benefits of these agents are attributed to osmotic diuresis, natriuresis, and improved vascular compliance [13]. Importantly, our findings reinforce that the antihypertensive effect of SGLT-2 inhibitors is evident in both diabetic and non-diabetic hypertensive patients, supporting evidence from the DECLARE-TIMI 58 and DAPA-HF trials [14], which demonstrated beneficial effects even in patients without diabetes.

Cardiovascular events and mortality

During follow-up, cardiovascular events were numerically higher in the diabetic group, including myocardial infarction (5% vs. 2%) and stroke (3% vs. 1%). Although these differences were not statistically significant, the trend reflects the well-established observation that patients with diabetes carry a two- to fourfold higher risk of adverse cardiovascular outcomes compared to non-diabetic individuals [14,15]. Similar patterns were noted in landmark trials, such as the EMPA-REG OUTCOME and CANVAS, where patients with diabetes demonstrated higher event rates but also derived significant cardiovascular protection from SGLT-2 inhibitors [16].

Hospitalization for heart failure occurred less frequently in the non-diabetic group (3% vs. 6%), and survival analysis showed better event-free survival in non-diabetic hypertensive patients. This finding is in line with the DECLARE-TIMI 58 and DAPA-HF trials, which showed that SGLT-2 inhibitors significantly reduce hospitalization for heart failure irrespective of diabetes status [17]. The consistent benefit observed in our study strengthens the hypothesis that the cardioprotective effects of these drugs are largely independent of glycemic control, potentially mediated by hemodynamic and myocardial energy metabolism effects [18].

All-cause mortality was also higher in the diabetic group (5% vs. 2%), although the difference was not statistically significant. A similar mortality benefit favoring SGLT-2 inhibitors was highlighted in the EMPA-REG OUTCOME trial, where empagliflozin significantly reduced cardiovascular and all-cause mortality in T2DM patients [19]. While our study was not powered to detect mortality differences, the trend towards improved survival in non-diabetics suggests that SGLT-2 inhibitors may have broader applications in high-risk hypertensive populations, extending beyond glycemic control.

Predictors of adverse cardiovascular events

Cox regression analysis in our study identified higher baseline HbA1c and longer duration of hypertension as independent predictors of adverse cardiovascular outcomes. These findings are consistent with prior evidence that poor glycemic control accelerates atherosclerosis, endothelial dysfunction, and left ventricular remodeling [20]. The Diabetes Control and Complications Trial and subsequent follow-ups have consistently shown that higher HbA1c levels are strongly linked with macrovascular complications [21]. Similarly, long-standing hypertension is a well-established risk factor for cardiovascular events due to cumulative vascular damage [22]. Importantly, the protective effect of SGLT-2 inhibitors against hospitalization for heart failure across both groups in our study supports mechanistic hypotheses regarding volume regulation, myocardial remodeling, and renal hemodynamic stabilization [23,24].

Strengths and limitations

This study provides real-world data on the cardiovascular outcomes of SGLT-2 inhibitors in hypertensive patients with and without diabetes, an area where evidence remains limited. The use of well-defined inclusion criteria and clinically relevant endpoints enhances its validity. However, the retrospective design and limited sample size restrict causal inference and may have underpowered the detection of differences in secondary outcomes such as myocardial infarction and stroke. Furthermore, residual confounding related to concomitant therapies cannot be excluded.

## Conclusions

In summary, our findings demonstrate that SGLT-2 inhibitors improve blood pressure control and reduce cardiovascular morbidity in hypertensive patients, with or without diabetes, although patients with diabetes continue to experience higher absolute event rates. These results align with evidence from major randomized trials and support the expanding role of SGLT-2 inhibitors as cardioprotective agents beyond glycemic regulation.

## References

[1] Gaziano TA (2022). Cardiovascular diseases worldwide. Public Health Approach to Cardiovasc Disease Prevention and Management.

[2] Roman G, Stoian AP (2021). Cardiovascular risk/disease in type 2 diabetes mellitus. Type 2 Diabetes - From Pathophysiology to Cyber Systems.

[3] Petrie JR, Guzik TJ, Touyz RM (2018). Diabetes, hypertension, and cardiovascular disease: clinical insights and vascular mechanisms. Can J Cardiol.

[4] Wilcox CS (2020). Antihypertensive and renal mechanisms of SGLT2 (sodium-glucose linked transporter 2) inhibitors. Hypertension.

[5] Cowie MR, Fisher M (2020). SGLT2 inhibitors: mechanisms of cardiovascular benefit beyond glycaemic control. Nat Rev Cardiol.

[6] Yu J, Zhou Z, Mahaffey KW (2021). An exploration of the heterogeneity in effects of SGLT2 inhibition on cardiovascular and all-cause mortality in the EMPA-REG OUTCOME, CANVAS Program, DECLARE-TIMI 58, and CREDENCE trials. Int J Cardiol.

[7] Chahade JJ, Kim R, Ussher JR (2021). Cardiovascular outcome trials in type 2 diabetes: food for thought. Future Cardiol.

[8] Ibrahim MO, Mohamed OM, Ali NM (2025). Sodium-glucose cotransporter 2 (SGLT2) inhibitors in non-diabetic heart failure: a systematic review of randomized controlled trials and real-world evidence. Cureus.

[9] Mazin I, Chernomordik F, Fefer P, Matetzky S, Beigel R (2022). The impact of novel anti-diabetic medications on CV outcomes: a new therapeutic horizon for diabetic and non-diabetic cardiac patients. Journal of Clinical Medicine.

[10] Lee B, Holstein-Rathlou NH, Sosnovtseva O, Sørensen CM (2023). Renoprotective effects of GLP-1 receptor agonists and SGLT-2 inhibitors-is hemodynamics the key point?. Am J Physiol Cell Physiol.

[11] Siam NH, Snigdha NN, Tabasumma N, Parvin I (2024). Diabetes mellitus and cardiovascular disease: exploring epidemiology, pathophysiology, and treatment strategies. Rev Cardiovasc Med.

[12] Bechmann LE, Emanuelsson F, Nordestgaard BG, Benn M (2024). SGLT2-inhibition increases total, LDL, and HDL cholesterol and lowers triglycerides: Meta-analyses of 60 randomized trials, overall and by dose, ethnicity, and drug type. Atherosclerosis.

[13] Blebea NM, Pușcașu C, Ștefănescu E, Stăniguț AM (2025). Diuretic therapy: mechanisms, clinical applications, and management. J Mind Medi Sci.

[14] Nilsson PM, Holm H, Magnusson M (2024). New antidiabetic agents for the treatment of heart failure in hypertensive patients. Hypertension and Heart Failure: Epidemiology, Mechanisms and Treatment.

[15] Kowalska K, Wilczopolski P, Buławska D, Młynarska E, Rysz J, Franczyk B (2022). The importance of SGLT-2 inhibitors as both the prevention and the treatment of diabetic cardiomyopathy. Antioxidants (Basel).

[16] Margonato D, Galati G, Mazzetti S, Cannistraci R, Perseghin G, Margonato A, Mortara A (2021). Renal protection: a leading mechanism for cardiovascular benefit in patients treated with SGLT2 inhibitors. Heart Fail Rev.

[17] Talha KM, Anker SD, Butler J (2023). SGLT-2 inhibitors in heart failure: a review of current evidence. Int J Heart Fail.

[18] Bell DS, Goncalves E (2021). Diabetogenic effects of cardioprotective drugs. Diabetes Obes Metab.

[19] Abdul-Ghani M, Del Prato S, Chilton R, DeFronzo RA (2016). SGLT2 inhibitors and cardiovascular risk: lessons learned from the EMPA-REG OUTCOME study. Diabetes Care.

[20] Ceriello A, Lucisano G, Prattichizzo F (2022). HbA1c variability predicts cardiovascular complications in type 2 diabetes regardless of being at glycemic target. Cardiovasc Diabetol.

[21] Lai YR, Huang CC, Chiu WC (2019). HbA1C variability is strongly associated with the severity of cardiovascular autonomic neuropathy in patients with type 2 diabetes after longer diabetes duration. Front Neurosci.

[22] Al-Kindi S, Nasir K, Rajagopalan S (2024). Elemental risk: role of metals in cardiovascular disease risk. J Am Coll Cardiol.

[23] Girardi AC, Polidoro JZ, Castro PC, Pio-Abreu A, Noronha IL, Drager LF (2024). Mechanisms of heart failure and chronic kidney disease protection by SGLT2 inhibitors in nondiabetic conditions. Am J Physiol Cell Physiol.

[24] Packer M (2022). Critical reanalysis of the mechanisms underlying the cardiorenal benefits of SGLT2 inhibitors and reaffirmation of the nutrient deprivation signaling/autophagy hypothesis. Circulation.

